# Diagnosis of latent tuberculosis infection among HIV discordant partners using interferon gamma release assays

**DOI:** 10.1186/1471-2334-11-264

**Published:** 2011-09-30

**Authors:** Naasha J Talati, Esteban Gonzalez-Diaz, Charles Mutemba, Joyanna Wendt, William Kilembe, Lawrence Mwananyanda, Elwyn Chomba, Susan Allen, Carlos del Rio, Henry M Blumberg

**Affiliations:** 1 Department of Medicine, University of Pennsylvania, Philadelphia, PA 19019, USA; 2 Department of Medicine, Hospital Civil de Guadalajara, Guadalajara 44280, Mexico; 3 Zambia Emory HIV Research Project, Lusaka, Zambia; 4 Department of Medicine, Emory University, Atlanta 30329, USA; 5 Rollins School of Public Health, Emory University, Atlanta 30329, USA

## Abstract

**Background:**

There is limited data on the effect of HIV status and CD4 counts on performance of Interferon-**g **Release assays (IGRAs) for diagnosis of latent tuberculosis infection (LTBI).

**Methods:**

A cross sectional study was conducted to assess the prevalence of and risk factors for a positive diagnostic test for LTBI, using tuberculin skin test (TST) and IGRAs among HIV-discordant couples in Zambia.

**Results:**

A total of 596 subjects (298 couples) were enrolled. Median CD4 count among HIV positive persons was 388 cells/μl, (range 51-1330). HIV negative persons were more likely than their HIV positive partner, to have a positive diagnostic test for LTBI with TST (203 vs 128), QFT (171 vs 109) and TSPOT (156 vs. 109). On multivariate analysis, HIV negative status was an independent predictor for a positive QFT (OR = 2.22, 95% CI 1.42- 3.46) and TSPOT (OR = 1.79, 95% CI 1.16-2.77). Among HIV positive subjects a CD4 count ≥ 388 cells/μl was associated with a positive TST (OR = 1.76 95% CI 1.10-2.82) and QFT (OR = 1.71 95% CI 1.06-2.77) but not TSPOT (OR = 1.20 95% CI 0.74-1.94).

**Conclusions:**

Persons with HIV had significantly fewer positive diagnostic tests for LTBI with TST, QFT and TSPOT. Persons with a CD4 count < 388 cells/μl were less likely to have a positive TST or QFT, but not less likely to have a positive TSPOT. TSPOT may perform better than TST or QFT in HIV positive individuals.

## Background

HIV and tuberculosis (TB) are the leading causes of death among adults due to an infectious disease worldwide. It is estimated that > 13 million people are co-infected with HIV and *Mycobacterium tuberculosis *[1]. The World Health Organization (WHO) estimates that there are approximately 9.3 million new cases of active TB and nearly 2 million deaths due to the disease worldwide each year [2,3]. Twenty-seven percent of TB cases and 31% of TB-related deaths occur in Africa, home to only 11% of the world's population [4].

HIV infection is the most important risk factor for progression from latent tuberculosis infection (LTBI) to active TB [5,6]. In patients with HIV and LTBI, the annual risk of progression to active TB is approximately 10% per year [7-9] compared to a lifetime risk of 5-10% in immunocompetent persons [7]. Diagnosis and treatment of LTBI is a major strategy for TB control and prevention in the US [7,10]. WHO has recommended the implementation of isoniazid preventive therapy for HIV-seropositive persons in an effort to prevent additional cases of TB, but this strategy has not yet been widely adopted in Africa [3].

For nearly a century, diagnosis of LTBI has relied on the tuberculin skin test (TST) which has several limitations including low specificity due to cross reaction with BCG vaccination and non-tuberculous mycobacteria (NTM) and low sensitivity in HIV infection. New diagnostic tests for tuberculosis are urgently needed to enhance global TB control [11,12].

Two Interferon-γ release assays (IGRAs) are now commercially available for the diagnosis of LTBI (QuantiFERON-TB Gold in Tube test [QFT] and T-SPOT.TB [TSPOT]) [13,14]. IGRAs provide increased specificity over TST, because the antigens used in the test do not cross react with BCG or other NTM. However, there is limited data on whether these tests provide any benefit over TST in immunocompromised individuals, such as those with HIV, particularly in high prevalence countries [14-21].

We conducted an observational cohort study to assess the performance of three diagnostic tests for LTBI (TST, QFT and TSPOT) in HIV positive persons and compared these results to the results of their HIV negative partner (control group), in Zambia. The goals of our study were: 1. To assess prevalence of a positive test for latent tuberculosis infection; 2. To assess concordance between TST, QFT and IGRAs; 3. To determine whether HIV is a risk factor for a positive test with TST, QFT or TSPOT; 4. Among HIV positive individuals to determine if CD4 count is an independent predictor for a positive TST, QFT or TSPOT.

## Methods

The study was conducted in Lusaka at the Zambia Emory HIV Research Project (ZEHRP). ZEHRP consists of a cohort of heterosexual HIV discordant couples and promotes couple's voluntary counseling and testing (CVCT) as a method of HIV prevention and as an entry-point into HIV clinical care [22]. Recruitment and study procedures have been described elsewhere [23-27].

HIV-infected individuals (> 16 years of age) and their discordant (HIV-seronegative) partner were offered an opportunity to enroll in the study. Exclusion criteria included pregnancy, active TB, or if the couple failed to come to the clinic appointment together. The study was approved by the Emory University Institutional Review Board (IRB) and the University of Zambia Research Ethics Committee and study subjects provided written informed consent.

Each study subject had two study visits. At the first study visit, study subjects were asked to complete a questionnaire that included questions regarding exposure to TB at home, history of BCG vaccination and history of incarceration. Blood was drawn from study participants for IGRA tests (3 ml for the QFT test [Cellestis Inc, Australia] test and 8 ml for the TSPOT [Oxford Immunotec, Oxford, UK]). HIV infected persons had a CD4 count performed. Following the blood draw, a TST was placed using 0.1 ml of PPD reagent (Tubersol, Sanofi Pasteur) by the Mantoux method [6]. Study participants were instructed to return in 48 to 72 hours to have the TST read. The amount of induration (in mm) was recorded by a trained health care provider. Based on national and international guidelines, a positive TST was defined as ≥ 5 mm of induration in HIV-infected persons and ≥ 10 mm in HIV-seronegative persons [7]. Persons with a positive TST had a chest radiograph performed and were questioned about symptoms in order to exclude active TB disease.

The QFT and TSPOT tests were performed based on the manufacturer's instructions and as previously reported [12-14,16]. Interferon-γ release was measured using ELISA after stimulation of sensitized T-cells using TB specific antigens. The test was considered positive if the interferon-γ response to TB antigens minus the negative control was ≥ 0.35 IU/ml and > 25% of the negative control; negative if these criteria were not met; and indeterminate if either the negative control had a result of ≥ 8 IU/ml, or if the positive control had a result of < 0.5 IU/ml of interferon-γ.

For TSPOT 250,000 peripheral blood mononuclear cells (PBMCs) were isolated and plated per well. Each test consisted of four wells: a negative control, a positive control (PHA) and TB specific antigens (CFP10 and ESAT 6). Spot forming units were counted manually and using an EliSpot Reader, (Cellular Technology Ltd., Cleveland, OH). A positive TSPOT test was defined as a response to either ESAT6 or CFP10 minus the negative control that is ≥ 8 spot forming cells and > 2 times the negative control; the test was negative if these criteria were not met; and the test was indeterminate if the reading in the negative control was > 20 spots or if the reading in the positive control were < 20 spots. All reported TSPOT results are based on results from the ELISPOT Reader.

### Sample size and statistical analysis

Sample size calculation was performed using prevalence of LTBI in Zambia from a previous study [28]. The precision method was used, by estimating the half width of the 95% confidence intervals we calculated a sample size of 300 couples.

Data analysis was performed using SAS 9.1 (SAS Inc, Cary, NC). Outcomes of interest included prevalence of LTBI, concordance between diagnostic tests, and risk factors associated with a positive test result. Concordance between the diagnostic tests for LTBI (IGRAs and TST) was measured using κ-statistic [13]. Risk factors for diagnostic test positivity were evaluated using odds ratios and 95% confidence interval. Variables that were analyzed included age, gender, HIV status, CD4 count, household income, exposure to a household member with TB within the last one month, history of incarceration and history of BCG vaccination. Initially univariate analysis was performed to assess risk factors for a positive test. Because of the matched study design a conditional logistic regression was performed to determine if HIV was a risk factor for a positive TST, QFT and TSPOT when comparing each HIV positive person to their HIV negative partner. Variables included in the final model were those that were significant on univariate analysis, had biological plausibility, were confounders or effect modifiers. A subset analysis was then performed on only the HIV positive persons using unconditional logistic regression to measure the association between CD4 count and a positive test result. Age and CD4 count were converted to categorical variables. Median values were used to convert continuous variables to categorical variables. We defined a p-value of < 0.05 as being statistically significant.

## Results

**Figure 1:**
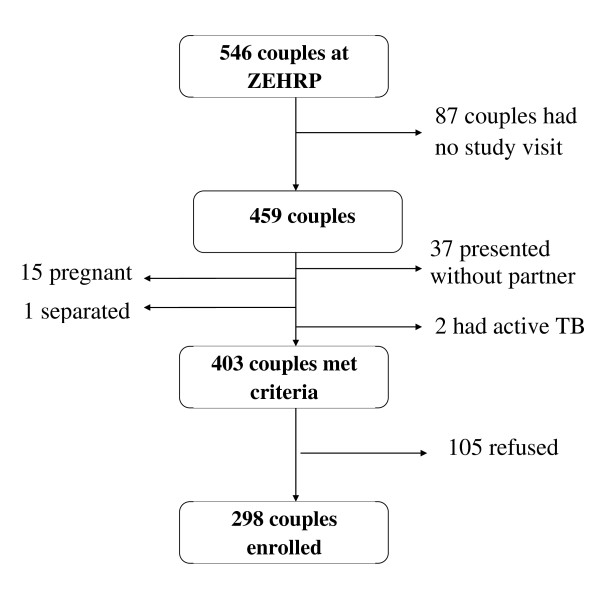
**Enrollment of Study Subjects from ZEHRP Cohort**.

Out of a cohort of 546 HIV-discordant couples currently enrolled at ZEHRP 403 couples met the prescreening criteria for study enrollment. The cross section for this study consists of 298 discordant couples or 596 study subjects (Figure 1). The median age of study participants was 33 years (range 17-59 years), median monthly income was the equivalent of US $32 (range US $ 0-1908). Women were significantly more likely to be HIV positive than men (60% vs 40%, p < 0.0001). Median CD4 count among HIV positive persons was 388 cells/μl, (range 51-1330 cells/μl). None of the study subjects were on antiretroviral therapy.

Demographic and clinical data for study subjects is summarized in Table 1.

**Table 1:** Clinical characteristics of HIV positive and HIV negative subjects enrolled in the study (n = 596)

Characteristics of study subjects	HIV positive n = 298	HIV negative n = 298	*p-value*
Age median and range	32 (18-59 years)	34 (17-59 years)	0.045
Male Gender	118 (40%)	180 (60%)	< 0.0001
History of prior incarceration	16 (5%)	18 (6%)	0.7
History of BCG vaccination	223(75%)	220 (74%)	0.9
History of household TB exposure in past 30 days	33 (11%)	37(12%)	0.8
Median monthy income $	32	32	0.6
Median CD4 count	388 cells/μl		

BCG: Bacillus Calmette-Guérin

HIV: Human Immunodeficiency Virus

### Diagnostic Test Results

#### Tuberculin Skin Test (TST)

A total of 331 (55.5%) persons had a positive TST, 252 (42.3%) had a negative TST, and 13 (2.2%) did not return to have their TST read. The median TST reading for persons with HIV was 18 mm and for persons without HIV was 16.5 mm (p-value = 0.05).

#### QuantiFERON-TB Gold in Tube (QFT) Test

280 (47.0%) persons had a positive QFT, 281 (47.2%) had a negative QFT and 35 (5.8%) had an indeterminate test. Among persons with a positive QFT, persons without HIV secreted a higher level of Interferon γ 3.5 IU/ml in comparison to people who had HIV 1.8 IU/ml (p-value = 0.0001).

#### TSPOT.TB (TSPOT) Test

A total of 265 (44.5%) had a positive TSPOT, 313 (52.5%) had a negative test, and 18 (3.1%) had an indeterminate result. Among persons with a positive TSPOT, HIV positive individuals exhibited 44.2 spots/ml and HIV negative persons had 53 spots/ml (p-value = 0.3).

HIV positive persons were significantly less likely to have a positive TST, QFT or TSPOT result (Table 2 ).

**Table 2:** Prevalence of a positive diagnostic test for Latent Tuberculosis Infection (LTBI) stratified by HIV status (n = 596)

Diagnostic Test for LTBI	HIV positive n = 298	HIV negative n = 298	p-value
TST	128 (43%)	203 (69%)	0.0003
QFT	109 (37%)	171 (58%)	0.006
TSPOT	109 (37%)	156 (53%)	0.005

TST: Tuberculin Skin Test

QFT: QuantiFERON-TB Gold In Tube Test

TSPOT: TSPOT.TB Test

HIV: Human Immunodeficiency Virus

### Indeterminate Test results with IGRAs

All 18 indeterminate TSPOT results occurred due to technical errors resulting in a high value in the negative control well. Five of the 35 indeterminate test results with the QFT occurred because of inadequate interferon-γ release in response to the positive control. All five of these cases occurred among HIV positive individuals and the median CD4 count was 264 cells/μl.

### Test results stratified by CD4 counts

**Figure 2:**
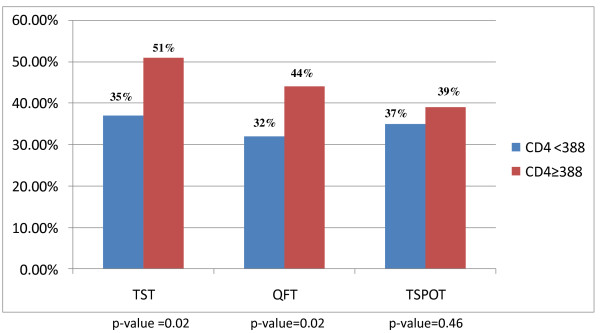
**Positive diagnostic test for latent tuberculosis infection stratified by CD4 count (n = 298)**.

Among HIV -seropositive individuals, subjects with a CD4 count < 388 cells/μl were less likely to have a positive test with TST and QFT, when compared to subjects with CD4 count ≥ 388 cells/μl. This difference was not seen with TSPOT (Figure 2).

### Concordance Between Diagnostic Tests for Latent TB Infection

Concordance between TST and QFT, TST and TSPOT and QFT and TSPOT was measured among HIV positive and HIV negative individuals. Overall concordance was moderate and there was no difference when comparing concordance between HIV positive and HIV negative subjects. (Table 3 ). For the TSPOT test, concordance between readings with a magnifying lens verses an ELISPOT reader was moderate κ = 0.67 (95% CI 0.61-0.73).

**Table 3:** Concordance between the three diagnostic tests described in all subjects, HIV seropositive subjects and HIV seronegative subjects

Test in HIV positive subjects (n = 298)	Agreement (%)	κ	95% Confidence Interval
TST vs QFT	75%	0.53	0.43, 0.64
TST vs TSPOT	76%	0.4	0.3, 0.52
TSPOT vs QFT	74%	0.37	0.26, 0.49
**Test in HIV negative subjects (n = 298)**	**Agreement (%)**	**κ**	**95% Confidence Interval**
TST vs QFT	72%	0.44	0.33, 0.55
TST vs TSPOT	70%	0.3	0.2, 0.4
TSPOT vs QFT	75%	0.46	0.35, 0.57

TST: Tuberculin Skin Test

QFT: Quantiferon-TB Gold In Tube Test

TSPOT: TSPOT.TB Test

HIV: Human Immunodeficiency Virus

### Risk factors for a positive diagnostic test for LTBI

Univariate analysis was performed and HIV negative status was found to be a risk factor for a positive test for TST(OR = 2.87, 95% CI = 1.94-3.97), QFT (OR = 2.18 95% CI = 1.48-2.99) and TSPOT (OR = 1.97 95% CI = 1.26-2.53). In addition males were more likely to have a positive TST (OR = 1.97 95% CI 1.39-2.79) and TSPOT (OR = 1.80 95% CI 1.27-2.55), however this effect was only seen on univariate analysis. BCG vaccination, household contact with a case of active TB, history of prison stay and income were not found to be risk factors for a positive test with TST, QFT or TSPOT. On multivariate analysis subjects who were HIV negative were more likely to have a positive TST, QFT and TSPOT, when controlling for age and gender. (Table 4) We found interaction between age and HIV in the multivariate model for tuberculin skin test. Among persons who are HIV negative, subjects who were < 32 years have an OR for a positive TST of 1.86; subjects who were > 32 years have an OR for a positive TST of 3.67.

**Table 4:** Multivariate Analysis, using Conditional Logistic Regression to evaluate Risk Factors for a Positive Diagnostic Test for Latent Tuberculosis Infection

Risk factors for positive TST	Odds Ratio	95% Confidence Interval
Age < 32 years	0.98	0.36-2.62
Male Gender	1.33	0.79-2.22
HIV negative* Age < 32 years Age ≥ 32 years	**1.86** **3.67**	**1.06-2.67** **1.60-8.39**
**Risk factors for positive QFT**	**Odds Ratio**	**95% Confidence Interval**
Age < 32 years	0.56	0.24-1.36
Male Gender	0.92	0.55-1.53
HIV negative	**2.22**	**1.42-3.46**
**Risk factors for positive TSPOT**	**Prevalence Ratio**	**95% Confidence Interval**
Age < 32 years	0.98	0.42-2.28
Male Gender	1.59	0.92-2.76
HIV negative	**1.79**	**1.16-2.77**

*Interaction between age and HIV

TST: Tuberculin Skin Test

QFT: Quantiferon-TB Gold In Tube Test

TSPOT: TSPOT.TB Test

HIV: Human Immunodeficiency Virus

We then performed multivariate analysis on HIV positive individuals alone and found that subjects with a CD4 count ≥ 388 cells/μl were more likely to have a positive TST (OR 1.76 95% CI 1.10-2.82) or QFT (OR 1.71, 95% CI 1.06-2.77). This association was not seen with TSPOT (OR1.20, 95% CI 0.74-1.94).

## Discussion

This is the first study to investigate all three diagnostic tests for latent tuberculosis infection (TST, QFT and TSPOT) in an HIV positive population and to compare results to an HIV negative partner as a control group. Using HIV discordant couples allowed us to compare study subjects with similar demographic, socioeconomic and exposure data to determine what the effect of HIV is on LTBI test positivity. The TST has been shown to have decreased sensitivity among HIV positive persons but there have been limited data on the sensitivity of IGRAs in this population [29-31]. Since one of the strategies to control tuberculosis is diagnosis and treatment of LTBI particularly among HIV-infected persons, we sought to assess IGRAs for the diagnosis LTBI among HIV-infected persons.

The prevalence of a positive test with TST, QFT and TSPOT was significantly lower among HIV positive persons, when compared to HIV negative persons, suggesting these tests do not perform as well in HIV positive persons. Induration with TST was greater in HIV negative than in HIV positive persons and amount of interferon-gamma release with QFT was greater among HIV negative persons than HIV positive persons. There was no difference in spot forming cells for TSPOT between HIV positive and HIV negative persons. TST and QFT produced more positive test results in HIV positive and negative individuals than TSPOT but this was not statistically significant.

On multivariate analysis being HIV negative was the only predictor for a positive test result, with TST, QFT and TSPOT, while controlling for other risk factors. A study from Africa compared QFT in HIV positive and negative subjects within the same population and was unable to show a difference in rates of test positivity based on HIV status. However that study had a small sample size and the HIV negative (controls) were not as closely matched as in our study [18]. A study from Spain did show that rates of positive QFT were lower among HIV positive than HIV negative individuals but TSPOT was not evaluated [32].

We performed multivariate analysis to assess whether CD4 count was an independent predictor of TST, QFT or TSPOT positivity. Among HIV-seropositive persons, a CD4 count ≥ 388 cells/μl was associated with a positive TST and QFT. CD4 count was not a predictor for a positive test result with TSPOT. This finding suggests that TSPOT may work better than TST or QFT among HIV positive individuals as results are not dependant on a patient's CD4 count. A study from Uganda also found that the rate of positive TSPOT results were comparable in HIV positive patients with CD4 counts < 100, 100-250 and > 250 cells/μl, however no multivariate analysis was performed in this study [33].

We compared concordance between TST and IGRAs, and between QFT and TSPOT and found fair concordance. There was no difference in concordance when we looked at just HIV-seropositive persons, or only HIV negative persons. Several other studies have shown fair to poor concordance in HIV [19-21]. Since concordance depends on prevalence, values from developed countries are lower than those from high incidence developing countries and are difficult to compare [14-18].

Most studies in HIV positive individuals have shown a large number of indeterminate results and these have been associated with low CD4 counts [16,17]. Our study had a low incidence of indeterminate test results and most of these occurred due to a high interferon reading in the negative control well, a finding that occurs due to technical problems. Five cases of indeterminate QFT results occurred due to inadequate interferon in the positive control. All of these occurred in HIV positive individuals. We had fewer indeterminate test results with TSPOT than with QFT.

Our study has several limitations. The median CD4 count in our population was high (388 cells/μl) and no study subject was on HAART. It is difficult to assess how these tests would perform at lower CD4 counts. Our study used the HIV negative partner as a control, to estimate the baseline prevalence of latent tuberculosis in the study population. We realize that both partners within a couple would not have identical exposures to tuberculosis, and that this may be considered a shortcoming of our study. However we do feel that their exposures would be similar and therefore both groups should have a similar rate of LTBI. Future studies could ask for more detailed exposure histories. Our study had a cross sectional design and therefore we don't have longitudinal data to determine whether a positive IGRA predicts future risk of active TB. However longitudinal studies are not practical due to the need for a large sample size and follow up over several years.

The advantage of conducting a study in a high incidence country is that we have a large number of positive IGRA test results and therefore have more power for analysis of risk factors. In addition, we used a unique study design where we matched each HIV positive person to their HIV negative domestic partner and then carried out a matched analysis using unconditional logistic regression. This is the first study to show that TSPOT may have an advantage over TST and QFT for diagnosis of LTBI among HIV positive persons.

## Conclusions

In conclusion, our study suggests that HIV positive status does decrease the sensitivity of TST, QFT and TSPOT. On multivariate analysis HIV status was the only predictor of test positivity for TST, QFT and TSPOT. Among HIV positive subjects a CD4 count > 388 cells/μl was associated with a positive TST and QFT, but not TSPOT. Based on the findings of our study it appears that TSPOT may be a better diagnostic test for LTBI in HIV positive persons, as results do not depend on CD4 count. Further research to evaluate use of TSPOT at lower CD4 counts and in other immunocompromised states is needed.

## Competing interests

The authors declare that they have no competing interests.

## Authors' contributions

NT was responsible for writing results and conclusions, EG was responsible for writing introduction and methods. CM contributed to study enrollment, study logistics, quality control, and methods. JW contributed to the methods and results section of the paper and was involved with study enrollment. WK was involved with patient recruitment and results, LM was involved in the results section. EC, SA, CR, HB were involved in study design, results and discussion. All authors read and approved the final manuscript.

## Pre-publication history

The pre-publication history for this paper can be accessed here:

http://www.biomedcentral.com/1471-2334/11/264/prepub
